# Clinical Effectiveness of Surgical Marginal Resection with Piezoelectric Device on Bisphosphonate-Related Osteonecrosis of the Jaws: A Retrospective Study

**DOI:** 10.3390/jcm14113792

**Published:** 2025-05-28

**Authors:** Claudia Manera, Martina Lee Tessari, Mariagrazia Boccuto, Christian Bacci

**Affiliations:** Department of Neurosciences, Section of Clinical Dentistry, University of Padova, 35121 Padova, Italy; claudia.manera@studenti.unipd.it (C.M.); martinalee.tessari@studenti.unipd.it (M.L.T.); mariagrazia.boccuto@studenti.unipd.it (M.B.)

**Keywords:** alveolar process, bisphosphonates, oral surgical procedures, osteonecrosis, surgical procedures, ultrasonic

## Abstract

**Background**: In 2020, the definition of Bisphosphonate-Related Osteonecrosis of the Jaws (BRONJ) was revised. The current definition is Medication-Related Osteonecrosis of the Jaws (MRONJ), to underline the fact that not only bisphosphonates are implicated in the onset of the disease. This study aims to investigate the efficacy of marginal resection using a piezoelectric device in patients with BRONJ. **Methods**: A retrospective study was conducted on subjects treated at the Dental Clinic University Hospital of Padua (Italy) from January 2017 to April 2024. Only patients diagnosed with BRONJ stages 1 and 2, who underwent marginal resection of the maxillae using a piezoelectric instrument were included. Patients who had received radiotherapy to the head and neck region, those with MRONJ, and those with primary tumors of the maxillary bones were excluded. Marginal resection was considered an effective treatment when complete epithelialization of the surgical site was achieved, with no signs or symptoms of disease, and the condition remained stable one-year post-operation. **Results**: In total, 21 patients (17 females and 4 males) were selected. A single resection was performed for each patient, resulting in a total of 21 surgeries: 14 in the mandible and 7 in the maxilla. At one-year post-surgery, 20 patients showed no signs or symptoms of the disease. One patient experienced two recurrences, both of which were subsequently treated. **Conclusions**: marginal resection using a piezoelectric device is an effective procedure for the treatment of BRONJ, although it remains a relatively invasive and destructive therapeutic approach.

## 1. Introduction

In 2020, the definition of Bisphosphonate-Related Osteonecrosis of the Jaws (BRONJ) was revised and redefined as an adverse drug-related reaction, characterized by the progressive destruction and necrosis of the mandibular and/or maxillary bone in individuals exposed for more than 8 weeks to antiresorptive drugs treatment, for which an increased risk of disease has been established, in the absence of prior head and neck radiotherapy [1,2]. The current definition of osteonecrosis is Medication-Related Osteonecrosis of the Jaws (MRONJ), to underline the fact that other drugs, not only bisphosphonates, are implicated in the onset of the disease [3,4]. Along with this new definition, the terms “major” and “minor” diagnostic criteria for BRONJ diagnosis have been eliminated [5]. The Italian Society of Oral Pathology and Medicine and the Italian Society of Maxillofacial Surgery (SIPMO-SICMF) have provided a list of clinical and radiographic signs and symptoms for the presentation of MRONJ, without distinguishing between major and minor criteria [6,7]. There have also been changes in the recommended treatments: the surgical approach, previously reserved for advanced stages of the disease, is now indicated and extended to less severe stages of MRONJ [8,9].

The pathophysiology of MRONJ is complex and multifactorial, involving suppression of bone turnover, infection, inflammation, and angiogenesis inhibition [10,11]. Recent studies have also highlighted the role of genetic factors in MRONJ susceptibility [12,13]. The incidence of MRONJ varies depending on the type and duration of antiresorptive therapy, with higher rates observed in cancer patients receiving high-dose intravenous bisphosphonates or denosumab [14,15].

Prevention strategies for MRONJ have been extensively studied and include dental screening before initiating antiresorptive therapy, maintaining good oral hygiene, and avoiding invasive dental procedures when possible [16,17]. When dental interventions are necessary, antibiotic prophylaxis and minimally invasive techniques are recommended [18].

In this new context, the surgical marginal resection intervention performed using piezoelectric instruments in MRONJ patients is introduced as a promising treatment option [19,20]. Piezoelectric surgery offers several advantages over traditional rotary instruments, including selective cutting of mineralized tissues, improved visibility, and reduced risk of soft tissue damage [21].

### Piezoelectric Devices in Medical–Dental Applications

Modern piezoelectric devices, widely used in the medical and dental fields, rely on the piezoelectric effect, discovered in 1880 by Pierre and Jacques Curie [22]. The inverse piezoelectric effect, discovered later by Gabriel Lippmann, also found applications in biomedical fields in the 2000s [23]. The piezoelectric effect occurs when mechanical deformation generates an electric charge in certain materials, while the inverse effect involves applying an electric field to cause mechanical deformation [24].

These devices use materials, typically crystalline ceramics, that deform when exposed to an electric current, generating vibration. This vibration is transferred to a working insert in devices that operate at frequencies between 24 and 40 KHz, commonly referred to as ultrasonic devices [25,26,27].

Ultrasonic piezoelectric devices are preferred in dental surgery due to their ability to precisely cut hard tissues, reducing the risk of damage to sensitive structures such as the inferior alveolar nerve or soft tissues like the gingiva and maxillary sinus membrane [28,29]. They can be used for procedures such as osteotomy, access to bone lesions, or foreign body removal [30,31,32]. The precision of these devices helps minimize damage, promotes faster bone healing, and results in less postoperative pain compared to traditional rotary tools [33,34]. It can be used in several clinical situations, such as the accidental displacement of foreign bodies into the maxillary sinus, to gain access to teeth or alveolar bone lesions, and to perform osteotomy in maxilla and mandible, such as in cases of bone lid surgery in posterior mandible [35,36].

Piezoelectric surgery also benefits from cavitation effects—when the vibrating insert interacts with irrigation solution, it forms micro-bubbles that help reduce bleeding and improve visibility during surgery [37]. These bubbles also have bactericidal properties and aid in cleaning bone debris [38]. Overall, piezoelectric devices are considered atraumatic, minimally invasive, and effective in enhancing surgical outcomes [39,40,41].

This paper aims to evaluate the effectiveness of marginal surgical resection with piezoelectric devices, as part of minimally invasive ultrasonic surgery, in the treatment of BRONJ. Specifically, the surgical procedure will be assessed in terms of healing and recurrence, in line with the prognostic indices present in the clinical-therapeutic recommendations of the SIPMO-SICMF [42].

## 2. Materials and Methods

This retrospective study reviewed medical records and histological examinations of 274 potentially eligible patients treated at the Dental Clinic of Padova from January 2017 to April 2024. This study focused on a series of BRONJ cases that were unresponsive to medical therapy and underwent marginal surgical resection using piezoelectric devices.

### 2.1. Inclusion and Exclusion Criteria

Table 1 summarizes inclusion and exclusion criteria.

For each patient, the following data were collected: gender, age, smoking status, comorbidities, BRONJ triggers, osteonecrosis resection site, bisphosphonate treatment indication, bisphosphonate type, and the presence of actinomyces in histological examinations.

**Figure 1:**
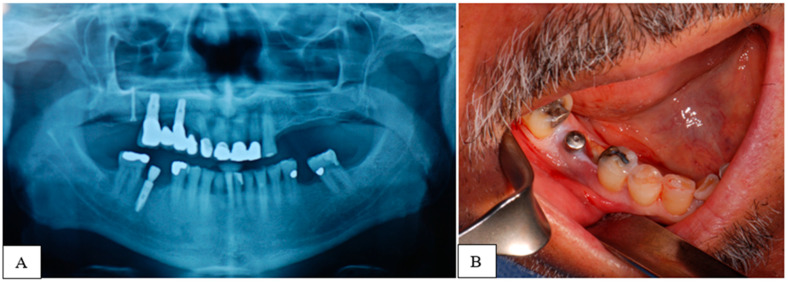
(**A**) Clinical case BRONJ stage 1b with osteonecrosis in the right mandible. Preoperative radiograph (T0). (**B**) Intra-oral clinical picture (T0) with distal bone exposure at second lower right premolar.

In this study, we used the SIPMO-SICMF staging, which differs in some details from the AAOMS staging system [43]. The corresponding stages are presented in Table 2 and Table 3. Presurgical (T0) clinical-radiographic signs and symptoms of BRONJ were recorded, as shown in Figure 1. From the orthopantomography (OPG), computed tomography (CT), or cone beam computed tomography (CBCT), the dimensions in millimeters of the lesion to be excised were predefined.

The surgical procedures were performed in a sterile environment, under conscious sedation, and by the same lead surgeon. After local anesthesia (Articaine Hydrochloride and Bupivacaine Hydrochloride), a mucoperiosteal flap was raised to expose the necrotic tissue. The osteotomy lines were made using a piezoelectric device and occasionally completed with a manual chisel. Finally, the tissue was excised. Intra-operatively, the surgical margins were adjusted, if necessary, based on the consistency, color, and bleeding of the bone tissue. The closure was achieved by first intention using resorbable sutures. Histological examination was requested for all cases.

**Figure 2:**
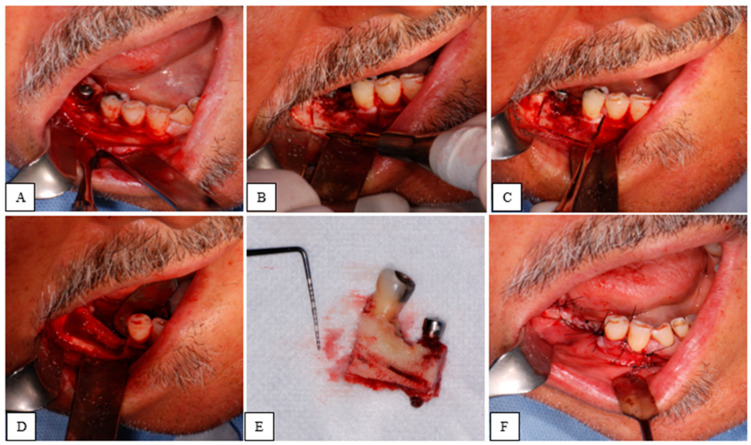
(**A**) Full thickness surgical flap detachment; (**B**) Piezoelectric device osteotomy lines; (**C**) Completion of osteotomy; (**D**) Clinical picture after resection; (**E**) Tissue removed with second lower right premolar and implant seat in first lower right molar position; (**F**) Suture and closure by primary intention.

All surgical procedures are presented in Figure 2.

Specifically, the marginal bone resection was performed using a piezoelectric device (PIEZOSURGERY^®^ touch, Mectron, Carasco, Genoa, Italy) set to bone mode with a high-frequency vibration of up to 36 kHz. The ultrasonic vibration tips required for the procedures were OT7, OT8L, OT8R, and OT12 for osteotomies, and OP1 for osteoplasty.

**Figure 3:**
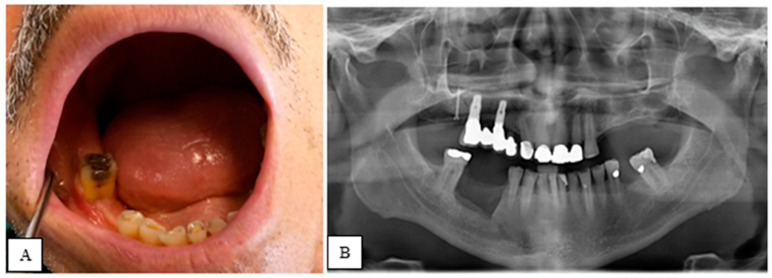
(**A**) Clinical picture 12 months after surgery (T3); (**B**) Radiography 12 months after piezoelectric surgery (T3).

As shown in Figure 3, in the post-operative period, the clinical signs and symptoms of BRONJ were reassessed, while radiographic evaluation was performed only at T2 and T3 to minimize the patient’s exposure to X-rays. For 9 days (3 days before and 6 days after the procedure), dual antibiotic therapy was prescribed: Amoxicillin and Clavulanic Acid (3 g/day) and Metronidazole (750 mg/day). In the post-operative phase, analgesic therapy (Paracetamol 3 g/day and Ibuprofen 1800 mg/day) and antiseptic therapy (0.2% chlorhexidine spray) were recommended to reduce pain and the microbial load in the oral cavity.

### 2.2. Success Criteria

The surgical marginal resection with a piezoelectric device was considered effective upon achieving the following criteria 1 year after surgery: absence of exposed bone in the oral cavity, complete epithelialization of the surgical site, and no clinical or radiographic signs or symptoms of BRONJ.

This study was conducted in accordance with the ethical principles outlined in the Helsinki Declaration and adhered to the Strengthening the Reporting of Observational Studies in Epidemiology (STROBE) guidelines for reporting observational studies [44,45]. The project was registered with the local ethics committee. Informed consent was obtained from all patients.

### 2.3. Statistical Analysis

A descriptive statistical analysis was performed on the data. Qualitative variables were presented as absolute frequencies and percentages, while quantitative variables were analyzed using the mean index. The data were analyzed and recorded using Excel 18.0-2021.

## 3. Results

Characteristics of all clinical cases are presented in Table 4 and Table 5 and summarize preoperative clinical and radiographic signs and symptoms, based on SIPMO-SICMF staging criteria.

A total of 21 patients were selected: 17 women (80.95%) and 4 men (19.05%). The average age was 74.38 years (range 63–86). In total, 9 patients (42.86%) were diagnosed with BRONJ stage 1, and 12 patients (57.14%) with BRONJ stage 2, based on the preoperative clinical and radiographic signs and symptoms and the SIPMO-SICMF staging criteria. Each stage was divided into asymptomatic (a) and symptomatic (b) cases. Regarding intra-oral triggers for osteonecrosis, the cause was not identified for 5 patients (23.81%). Overall, 7 patients (33.32%) were treated with bisphosphonates for osteometabolic reasons, mainly osteoporosis, and 14 patients (66.67%) for oncological reasons: 11 patients (52.38%) had bone metastases and 3 patients (14.29%) had Multiple Myeloma. Zoledronic acid (ZOL) had been prescribed for 15 patients (71.43%), Alendronate (ALE) for 4 patients (19.05%), and Ibandronate (IBA) for 2 patients (9.52%).

The most common comorbidities were hypertension, presented in 12 patients (57.14%), familial hypercholesterolemia presented in 5 patients (23.81%), and type II diabetes mellitus presented in 3 patients (14.29%). Other conditions, such as chronic kidney failure, were observed in less than 10% of the cases. In total, 10 patients (47.62%) were smokers.

Each patient underwent a single marginal resection, with a total of 21 surgeries performed: 14 resections (66.67%) were carried out on the mandible, and 7 resections (33.33%) on the maxilla. The operating time was found to be on average 48.81 min (with a standard deviation of 8.98 min). Values are listed in Table 6. All patients received histological confirmation of BRONJ, and in 9 samples (42.86%), actinomyces were found.

Table 7 presents clinical cases and corresponding clinical-radiographic signs and symptoms of BRONJ observed after piezoelectric marginal resection at follow-up (T1-T2-T3).

At the first follow-up (T1), 5 patients (23.81%) showed incomplete epithelialization of the surgical site, and other symptoms (trismus, lip dysesthesia) were present, although with reduced severity compared to the preoperative state. At 6 months post-resection (T2), in 1 patient (4.76%) (N°1), clinical and radiographic signs such as incomplete epithelialization, dysesthesia, and thickening of the trabecular bone were noted. At T3, the last follow-up, the same patient (N°1) showed worsening of the clinical picture with exposed bone in the oral cavity, lip dysesthesia, pain, and focal osteosclerosis. Pain was measured using the Visual Analogue Scale (VAS) and recorded a score of 4. This patient underwent two additional marginal resections using the piezoelectric device. For the following 3 years after the last resection, the patient was followed up biannually, with no further clinical or radiographic signs of BRONJ being observed.

## 4. Discussion

In this case series, the most frequent site of osteonecrosis was the mandible (66.67%). This result aligns with the literature, where the mandible is reported to be more commonly affected by BRONJ compared to the maxilla. In one study, the mandible was affected in 70% of cases, and in another study, in 60% [46,47]. The most widely accepted hypothesis is that the mandible is more susceptible to osteonecrosis due to its terminal vascularization [48]. The average age of patients at the time of surgery was 74.38 years (range 63–86), indicating that BRONJ tends to manifest in the 60–80 age group. This finding is consistent with numerous other studies, where the mean age ranges from 60 to 77 years [49,50,51]. One systematic review reports a mean age of 66.5 ± 4.7 years [52]. While the majority of patients fall within this age range, the onset of the primary condition and the initiation of bisphosphonate therapy may play a role in the age of BRONJ manifestation, as some studies report a mean age of 55.4 years [53].

Women represent most patients (80.95%), which is consistent with the higher incidence of BRONJ in women. Some studies suggest a gender ratio of approximately 3:1. This could be attributed to women’s longer life expectancy, the rising incidence of breast cancer, and the menopausal condition, which leads to a decrease in estrogen levels and consequently reduced bone mass. Regarding the primary condition for which bisphosphonates were prescribed, it can be said, in agreement with the international literature, that oncological patients develop BRONJ more frequently than osteometabolic patients [54]. The different potency of the drug, the route of administration, and the duration of bisphosphonate therapy all affect the outcomes. The role of periodontal disease as a risk factor for BRONJ, and more generally for inflammatory and infectious conditions in the oral cavity, has been extensively discussed [55]. Inflammation may induce bone necrosis both through the release of chemical mediators and via indirect action through edema, leading to reduced blood supply to the bone and subsequent necrosis [56].

Extraction surgery and implant therapy can connect the bone with the oral flora and induce trauma in tissues with altered metabolism and healing [57,58,59,60]. Poorly adjusted prosthetics also pose a local risk factor, as ill-fitting prostheses can exert excessive pressure, leading to thinning of soft tissues, ulceration, and bone exposure [61,62]. For 5 patients in this study (23.81%), no identifiable trigger for BRONJ was found. These cases may be due to anatomical predispositions or what is known as “spontaneous” drug-related ONJ, which may be associated with the patient’s pharmacogenetics, though there is insufficient evidence on this [63,64].

It is well-established that smoking causes vasoconstriction of blood vessels, leading to reduced blood flow to the bone and necrosis [65]. It also impairs wound healing, delaying the entire process [66]. In this study and in a similar paper, however, smoking did not seem to influence the surgical outcome, which might be due to patients either abstaining from smoking or reducing their daily cigarette consumption during the postoperative period [67].

In addition to the primary condition, comorbidities were observed, including hypertension, diabetes, and chronic kidney failure. The latter is considered a systemic risk factor for BRONJ, as excessive calcium excretion and inadequate renal reabsorption can disrupt calcium metabolism, also affecting the maxillary bones. Regarding hypertension, some studies have identified a correlation between high blood pressure and increased BRONJ risk, though the exact causal relationship remains unclear [68]. For diabetes, the literature does not yet provide a clear pathophysiological mechanism linking it to BRONJ onset. However, some studies suggest that microvascular damage caused by diabetes may also impact the bone tissue [69,70].

The histological confirmation of BRONJ was obtained for all cases, and the presence of actinomycetes was detected in the biopsy sample of 9 patients (42.86%), indicating superinfection of the necrotic bone.

The bacteria found in BRONJ are typically present in the oral cavity or are found in odontogenic and periodontal diseases. The most frequent pathogen in cases of bisphosphonate-induced osteonecrosis is, therefore, the actinomycete [71,72,73].

Piezoelectric instruments operate through the mechanical deformation of internal crystalline ceramics induced by an electric current. This deformation generates vibrations in the ultrasonic range, typically between 24 and 40 kHz, which are transmitted to the active tip of the ultrasonic insert.

The application of ultrasonic inserts in surgical procedures offers enhanced selectivity in cutting mineralized tissues, thus improving the preservation of surrounding soft tissues, particularly critical anatomical structures such as nerves and vessels. As noted by Bennardo et al. [74], the main limitation of piezoelectric instruments is the increased operative time compared to conventional osteotomies performed with rotary burs. However, the superior cutting selectivity enables cleaner and more precise osteotomies, as confirmed by Blaskovic et al. [75].

The literature presents conflicting evidence regarding the impact of piezoelectric devices on intraoperative bleeding. The cavitation effect generated by ultrasonic vibrations has been suggested to reduce bleeding and improve the surgical field’s visibility, as reported by Schlee et al. [37]. In contrast, a pilot study by Bennardo et al. found no statistically significant difference in bleeding compared to conventional osteotomy techniques [74]. Similarly, Walia et al. reported comparable levels of intraoperative bleeding during third molar extractions performed with piezoelectric and traditional methods [76].

Blaskovic et al. investigated bone healing in rats following osteotomies performed using three different methods: rotary bur, piezosurgery, and erbium laser. The study demonstrated that initial bone formation in defects prepared by piezosurgery was the most rapid, suggesting a potential advantage in postoperative bone regeneration [75].

Furthermore, Rocco et al. found that piezoelectric osteotomy was associated with significantly reduced postoperative pain and edema compared to traditional bur techniques [77]. The incidence of nerve injury was also lower in the piezoelectric group. Despite the greater safety profile concerning neural structures, the technique requires cautious application, particularly in anatomically complex regions [78].

Regarding the treatment of Medication-Related Osteonecrosis of the Jaws (MRONJ), current evidence suggests that surgical intervention is more appropriate than medical therapy in patients with advanced stages of the disease. Conversely, conservative management appears to produce favorable outcomes in asymptomatic patients with early stage MRONJ, as highlighted by Saluki et al. [79]. A conservative surgical approach, when combined with various adjuvant non-invasive therapies, such as ozone therapy, low-level laser therapy (LLLT), or the use of autologous blood-derived products combined with Nd:YAG laser, has demonstrated partial or complete healing across all disease stages, indicating its potential as a viable therapeutic option for MRONJ [80,81].

At T0, the clinical and radiographic manifestation of BRONJ in the selected cases aligns with what is reported in the literature: in no cases were symptoms and/or signs found that differed from those already known. Pain, a frequent and debilitating component in BRONJ patients, was reported by approximately one-third of the patients (28.57%) [82]. The results indicate that, one year after surgery (T3), 95.24% of the treated cases meet the success criteria established by this study to evaluate the effectiveness of piezoelectric marginal resection in BRONJ patients: 20 patients, in fact, showed complete epithelialization of the surgical site and absence of clinical and radiographic signs and symptoms of the disease.

However, to date, no comparative studies are available assessing the efficacy of resective osteotomy performed using traditional techniques versus piezosurgery. Nonetheless, similar case series show results comparable to those of this research. In these two studies, a total of 29 piezoelectric marginal resections were performed in BRONJ patients, both oncological and osteometabolic. These studies suggest that ultrasonic resection provides positive clinical outcomes in the treatment of bisphosphonate-induced osteonecrosis of the jaws [83,84]. Another case series monitored 6 BRONJ patients who underwent a single piezoelectric marginal resection for a period of 60 months, without observing signs or symptoms of the disease in the long term: 5 years after ultrasound surgery, no recurrences were reported [85].

At T3, only one patient (4.76%) presented, in addition to the persistence of the symptoms and signs recorded at T2, a worsening of the clinical condition. This clinical case was successfully re-treated with two additional piezoelectric marginal resections, and 4 years after the last surgery, no further signs or symptoms of BRONJ were observed.

The evaluation of postoperative bleeding is absolutely complex as closed drains are not positioned or positionable which allow the bleeding to be quantified in terms of cubic centimeters. However, evaluation of any post-operative bleeding, following the classification most commonly used in the literature, i.e., absence of bleeding, moderate bleeding, profuse bleeding that required something more than local haemostatic measures such as re-intervention, transfusion, or endotracheal intubation, was evaluated. None of these patients experienced post operative bleeding, according to the classification report before [86,87,88].

While this paper analyzes and describes an effective resective technique, the most effective approach to ONJ remains preventive [89]. Furthermore, other surgical approaches besides bone resection by piezosurgery can be taken into consideration, as also reported by Grzegorz Dawiec et al. [90].

The strength of this paper is to purpose a safe technique to perform surgical treatment of this pathology.

A limitation, the weakness of the research is represented by the sample size, which can be considered adequate in relation to study design but relatively small compared to larger cohorts of subjects. There are also limitations related to the evaluation of the study variables and the retrospective data collection. It would be useful to perform a case control study with, for example, other surgical techniques. In this sample, surgery was performed in mild sedation and local anesthesia [91]. This case series was conducted in a single hospital facility, and the patients were selected only from those managed by a single medical team. Another limitation is the relatively short 12-month post-operative follow-up period.

The future direction is to expand the case study and record the data in a prospective study. In the future, topical healing promoters could also be used [92].

## 5. Conclusions

This study demonstrates that piezoelectric marginal resection is effective in the treatment of BRONJ, although it remains an invasive procedure. Further studies are needed to include larger cohorts of patients. Additionally, there is hope for the future development of more efficient piezoelectric devices in order to establish this type of surgery as the “Gold Standard” for drug-related osteonecrosis.

## Figures and Tables

**Table 1:** Inclusion and exclusion criteria.

Inclusion Criteria	Exclusion Criteria
- Adult patients who took only bisphosphonates, not other MRONJ-related drugs, with BRONJ stages 1 and 2, undergoing marginal resection with piezoelectric devices - Patients followed by the same surgeon for both visits and surgery - Availability of clinical documentation - Patients underwent three follow-up visits at 1 month (T1), 6 months (T2), and 12 months (T3) post-surgery	- Patients who had undergone head and neck radiotherapy - Patients who took non-bisphosphonates drugs and developed MRONJ - Patients with primary or metastatic neoplasms of the maxillary bones

**Table 2:** Clinical and radiographic stages of Medication-Related Osteonecrosis of the Jaw (MRONJ) based on SIPMO-SICMF staging criteria [1].

Stage	Clinical Signs and Symptoms	CT Signs
Stage 1—Focal Mronj The presence of at least 1 clinical sign/symptom and increased bone density limited to the alveolar process at CT, with or without additional radiological signs. - Stage 1a: Asymptomatic (without pain) - Stage 1b: Symptomatic (the presence of pain and/or purulent discharge)	Abscess, bone exposure, halitosis, intraoral fistula, jaw pain of bone origin, mucosal inflammation, non-healing post-extraction socket, soft tissue swelling, spontaneous loss of bone fragments, sudden dental/implant mobility, purulent discharge, toothache and trismus.	Trabecular thickening and/or focal bone marrow sclerosis, with or without cortical erosion, osteolytic changes, thickening of the alveolar ridge, thickening of the lamina dura, persistent post-extraction socket, periodontal space widening, thickening of the inferior alveolar nerve canal, sequester formation.
Stage 2—Diffuse Mronj The presence of at least 1 clinical sign/symptom and increased bone density extending to the basal bone at CT, with or without additional radiological signs. - Stage 2a: asymptomatic (without pain) - Stage 2b: symptomatic (presence of pain and/or purulent discharge)	Same as Stage 1, plus mandibular deformation and numbness of the lips.	Diffuse bone marrow sclerosis, with or without cortical erosion, osteolytic changes, thickening of the alveolar ridge, thickening of the lamina dura, persistent post-extraction socket, periodontal space widening, thickening of the inferior alveolar nerve canal, sequester formation, periosteal reaction, and opacified maxillary sinus.
Stage 3—Complicated Mronj The presence of at least 1 clinical sign/symptom and increased bone density extended to the basal bone at CT, plus one or more of the following - Stage 3a: asymptomatic (without pain) - Stage 3b: symptomatic (presence of pain and/or purulent discharge)	Cutaneous fistula, mandible fracture, fluid discharge from the nose.	Osteosclerosis of adjacent bones (zygoma and hard palate), pathologic fracture, osteolysis extending to the maxillary sinus, sinus tract (oroantral, oronasal fistula, oro-cutaneous).

**Table 3:** Clinical and radiographic stages of Medication-Related Osteonecrosis of the Jaw (MRONJ) based on AAOMS staging criteria [43].

Stage	Symptoms	Clinical Findings	Radiographic Findings
Stage 0	- Odontalgia not explained by an odontogenic cause. - Dull, aching bone pain in the jaw, which may radiate to the temporomandibular joint region. - Sinus pain, which may be associated with inflammation and thickening of the maxillary sinus wall. - Altered neurosensory function.	- Loosening of teeth not explained by chronic periodontal disease. - Intraoral or extraoral swelling.	- Alveolar bone loss or resorption not attributable to chronic periodontal disease. - Changes to trabecular pattern sclerotic bone and no new bone in extraction sockets. - Regions of osteosclerosis involving the alveolar bone and/or the surrounding basilar bone. - Thickening/obscuring of periodontal ligament (thickening of the lamina dura, sclerosis, and decreased size of the periodontal ligament space).
Stage I	- Asymptomatic	- Exposed and necrotic bone or fistula that probes to the bone. - No evidence of infection/inflammation.	- May present with radiographic findings mentioned for Stage 0 that are localized to the alveolar bone region.
Stage II	- Symptomatic	- Exposed and necrotic bone, or fistula that probes to the bone. - Evidence of infection/inflammation.	- May present with radiographic findings mentioned for Stage 0 localized to the alveolar bone region.
Stage III	- Symptomatic	- Exposed and necrotic bone or fistulae that probes to the bone. - Evidence of infection. - One or more of the following: ○ Exposed necrotic bone extending beyond the region of alveolar bone (i.e., inferior border and ramus in the mandible, maxillary sinus, and zygoma in the maxilla). ○ Extraoral fistula. ○ Oral antral/oral–nasal communication.	- May be present: ○ Pathologic fracture. ○ Osteolysis extending to the inferior border of the mandible or sinus floor.

**Table 4:** Distribution of patients based on clinical signs and symptoms of Bisphosphonate-Related Osteonecrosis of the Jaw (BRONJ) recorded preoperatively (T0).

Clinical Signs and Symptoms of BRONJ (T0)	N° of Patients	%
Exposed bone	21	100
Halitosis	11	52.38
Dental mobility	7	33.33
Pain	6	28.57
Trismus	5	23.81
Failure of post-extraction alveolar mucosa repair	4	19.05
Soft tissue swelling	3	14.29
Lip paresthesia/dysesthesia	3	14.29
Implant mobility	2	9.52
Suppuration	2	9.52

**Table 5:** Distribution of patients based on radiographic signs of Bisphosphonate-Related Osteonecrosis of the Jaw (BRONJ) recorded preoperatively (T0).

Radiographic Signs of BRONJ (T0)	N° of Patients	%
Diffuse osteosclerosis	6	28.57
Focal medullary osteosclerosis	5	23.81
Widening of the periodontal space	5	23.81
Persistence of post-extraction alveolus	4	19.05
Sinusitis	4	19.05
Thickening of the alveolar canal	2	9.52
Oro-antral fistulas	2	9.52
Periosteal reaction	2	9.52

**Table 6:** Operative time values evaluated in minutes for each patient, mean, median, and standard deviation evaluation in minutes.

No. of Patients	Percentage (%)	Operative Time (min)	Mean (min)	Median (min)	Standard Deviation (min)
Total (21 patients)	100%		48.81	45	8.98
1	4.76%	75			
3	14.29%	60			
3	14.29%	55			
9	42.86%	45			
5	23.81%	40			

**Table 7:** Clinical cases and corresponding clinical-radiographic signs and symptoms of BRONJ observed after piezoelectric marginal resection at follow-up (T1-T2-T3).

N° Patient	T1 Clinical Signs and Symptoms of BRONJ	T1 Radiographic Signs of BRONJ	T2 Clinical Signs and Symptoms of BRONJ	T2 Radiographic Signs of BRONJ	T3 Clinical Signs and Symptoms of BRONJ	T3 Radiographic Signs of BRONJ
1	Incomplete epithelialization, Dysesthesia	X-rays not performed	Incomplete epithelialization, Dysesthesia	Trabecular thickening	Exposed bone, Dysesthesia, Pain	Focal medullary osteosclerosis
8	Incomplete epithelialization	X-rays not performed	Absent	Absent	Absent	Absent
13	Incomplete epithelialization, Trismus	X-rays not performed	Absent	Absent	Absent	Absent
14	Incomplete epithelialization	X-rays not performed	Absent	Absent	Absent	Absent
19	Incomplete epithelialization, Lip Dysesthesia	X-rays not performed	Absent	Absent	Absent	Absent

## Data Availability

Data are available on demand.

## Abbreviations

The following abbreviations are used in this manuscript:

OPG	Orthopantomography
CBCT	Cone Beam Computed Tomography
CT	Computed Tomography
ZOL	Zoledronic acid
ALE	Alendronate
IBA	Ibandronate
